# Evaluation of Perioperative Complications in the Management of Biliary Atresia

**DOI:** 10.3389/fped.2020.00460

**Published:** 2020-08-28

**Authors:** Min Du, Junfeng Wang, Yue Tang, Jingying Jiang, Gong Chen, Yanlei Huang, Zhen Shen, Rui Dong, Shan Zheng

**Affiliations:** Department of Pediatric Surgery, Children's Hospital of Fudan University, and Shanghai Key Laboratory of Birth Defect, Shanghai, China

**Keywords:** biliary atresia, glucocorticoid, jaundice clearance, native liver survival, perioperative complication

## Abstract

**Purpose:** To analyze the influence of perioperative complications in the management of biliary atresia (BA).

**Methods:** A retrospective study was performed using a total of 422 BA patients who underwent Kasai portoenterostomy (KPE) in a single institution between February 2016 and May 2017. Data on patients' clinical characteristics, laboratory examinations, perioperative complications, and outcomes were collected. Unpaired two-tailed *t*-test and χ^2^ test were employed for the comparison between BA patients with and without perioperative complications. Cox regression analysis was used to screen the risk factors for 2-years NLS in BA, and their influence on the 2-years NLS was analyzed using Kaplan–Meier survival analysis as well as the log-rank test.

**Results:** The incidence of perioperative complications, 6-months jaundice clearance (JC) and 2-years native liver survival (NLS) rate were 60.4, 59.5, and 56.6%, respectively. Patients with perioperative complications had lower serum albumin (ALB) level, but higher aspartate aminotransferase-to-platelet ratio index (APRI) and international normalized ratio (INR) levels when compared with those without perioperative complications (ALB, *P* < 0.05; APRI, *P* < 0.01; INR, *P* < 0.05). Moreover, perioperative complications were correlated with glucocorticoid administration (*P* = 0.002). Univariate Cox regression analysis showed no relationship between perioperative complications and 2-years NLS (*P* > 0.05). However, multivariate Cox regression analysis indicated 6-months JC was an independent protective factor for 2-years NLS [*P* < 0.0001, hazard ratio (HR) = 0.074, 95% confidence interval = 0.05–0.11], and concordance index of this prediction model including age, weight, APRI, glucocorticoid, and 6-months JC was 0.811.

**Conclusion:** Although perioperative complication is common during and after KPE, it had no influence on the prognosis of BA. However, assessment of the serum level of total bilirubin after KPE may serve as an important predictor for the outcome in BA.

## Introduction

Biliary atresia (BA) is a life-threatening pediatric hepatobiliary disease that is caused by a progressive inflammatory and fibrotic obliteration of the bile ducts (1). The pathophysiology of BA is not yet clearly understood, and theories on the pathogenesis of BA include immunological factors, genetic predisposition, environmental toxins, and infection (2). In Asia, the BA incidence (1/5,000–8,000 live births) is considerably higher compared with that in Europe (1/15,000–20,000 live births) (3, 4). According to the anatomical structure, the Japanese Society of Pediatric Surgery classified BA into three types, including type I BA (choledochal atresia), type II BA (the common hepatic duct atresia), and type III BA (hepatic portal atresia) (5). Type III BA was previously considered as the “uncorrectable” type with poor outcomes affecting 80–95% of patients (6, 7).

When left untreated, BA patients will die of cirrhosis and hepatic failure within 2 years (8). Kasai portoenterostomy (KPE) is considered the first-line treatment for BA and restores bile flow from the liver to the intestine (9). In previous studies, it was demonstrated that native liver survival (NLS) rates ranged from 20 to 76% at 1–3 years following KPE (10). If KPE fails, liver transplantation is the definitive therapeutic option (11). Even in cases of successful KPE, complications occur in ~60% of patients; however, large cohort studies that focus on the comprehensive perioperative complications of BA are limited (12, 13). Perioperative complications have previously been defined as complications that occurred during and up to 30 days after surgery (14). Pressing questions for surgeons to answer are as follows: What are the characteristics of perioperative complications of BA? And what is the impact of perioperative complications on the outcome of BA? This study aimed to characterize perioperative complications of BA and analyze the predictors of prognosis, and the relationship between perioperative complications and prognosis in BA.

## Patients and Methods

### Establishment of the Retrospective Cohort

This study was a retrospective study that was performed in Children's Hospital of Fudan University (Shanghai, China). The study was conducted in compliance with the Declaration of Helsinki and was approved by the institutional ethical review board. As type III BA was the most common and prone to poor outcomes, this study focused on type III BA. A total of 422 type III BA patients were enrolled. The follow-up endpoints were as follows: (1) 24 months after KPE; (2) patients passed away or underwent liver transplantation. Inclusion criteria were as follows: (1) surgical exploration and cholangiography that confirmed type III BA; (2) KPE was performed by the same operation team. Exclusion criteria were as follows: (1) patients who suffered from immunodeficiency, hemolytic jaundice, and life-threatening serious diseases involving other systems such as heart failure, respiratory failure, and kidney failure, and so on; (2) follow-up was <24 months; (3) data on perioperative complications and 2-years outcome were unavailable.

### Operative Technique and Postoperative Care

The KPE procedure adopted the classic Kasai procedure with spur valve for antireflux, and the length of the jejunal Roux-en-Y loop was 30–35 cm. Unified postoperative adjuvant therapies included antibiotics to prevent cholangitis (first 2 weeks after KPE: intravenous cefoperazone sulbactam, 100 mg/kg/d, every 8 h, and ornidazole 20 mg/kg/d, every 12 h; from 3 weeks to 6 months after KPE: sulfamethoxazole/trimethoprim, 25 mg/kg/d, every 12 h, and cefaclor, 20 mg/kg/d, every 8 h, are taken alternately every 2 weeks), ursodeoxycholic acid (20 mg/kg/d, twice a day), drugs of liver protection (glycyrrhizin, 25 mg/d, twice a day), fat-soluble vitamins (vitamin A and D drops, 2,000 IU/d, every day; vitamin E 10 mg/d, every day; vitamin K_1_, 5 mg/time, biweekly), and nutritional support. When insufficient bile flow was presented with gray-pigmented stool without any surgery-related complications, glucocorticoid (methylprednisolone starting at 4 mg/kg/d and tapering down for 3 months) was used. Jaundice clearance (JC) was defined as total bilirubin (TBIL) <20 μmol/L (15, 16).

### Data Collection

Baseline data were collected from each patient at the time of KPE, including gender, age, weight, the presence of cytomegalovirus (CMV) infection [immunoglobulin M (IgM^+^)], and extrahepatic malformations. The use of glucocorticoids and perioperative complications were acquired during and up to 30 days post-KPE. Perioperative complications were classified into surgical complications (SCs) and disease-related complications (DCs). Surgical complications were defined as complications that were directly related to surgery, whereas DCs were defined as complications that were related to the underlying disease (17, 18). Disease-related complications included symptoms that were absent pre-KPE, but occurred after KPE, and can be classified as liver dysfunction (hypoalbuminemia, coagulopathy), ascites, cholangitis, portal hypertension (esophageal variceal bleeding), and intrahepatic biliary lakes (hepatic cyst) (18). Serum albumin (ALB) <30 g/L was considered hypoproteinemia (19). Abnormal coagulation was judged by the presence of prolonged prothrombin time (PT) or activated partial thromboplastin time (APTT), or higher international normalized ratio (INR) or decreased fibrinogen (FIB) (reference ranges: PT, 11–14.5 s; APTT, 26–40 s; INR, 0.8–1.2; FIB, 2–4 g/L). Cholangitis was diagnosed upon clinical symptoms (unexplained fever, recurrent jaundice, and acholic stools) and laboratory examinations (elevated white blood cell counts and C-reactive protein, and higher bilirubin levels) (20). Ascites and hepatic cyst were found by the abdominal ultrasonography post-KPE. From each patient, laboratory examinations were collected at KPE including TBIL, direct bilirubin (DBIL), ALB, aspartate aminotransferase, alanine aminotransferase, γ-glutamyl transpeptidase (GGT), total bile acid, aspartate aminotransferase-to-platelet ratio index (APRI), hemoglobin, and INR. The above parameters were reevaluated by collecting medical records during hospitalization and outpatient clinical records or telephone communication after discharge up to 30 days after KPE. The follow-up data included JC and NLS at 1 month, 3 months, 6 months, 1 year, and 2 years after KPE. Among them, 2-years NLS was set as the main prognostic indicator in this study.

### Statistical Analysis

Statistical analyses were performed using SPSS software version 23.0 (IBM Corp., Armonk, NY, USA) and R software 3.6.0 (Lucent Technologies, Murray Hill, NJ, USA). Continuous data are presented as the mean ± standard deviation, and categorical variables are presented as absolute numbers and percentages. Unpaired two-tailed *t*-test and χ^2^ test were employed for the comparison between BA patients with and without perioperative complications. Univariate Cox regression analysis was employed to choose the potential predictive factors of 2-years NLS of BA (*P* < 0.2), and then the prediction model was established by the above factors and was analyzed using multivariate Cox regression analysis. Results were expressed as the hazard ratio (HR) and 95% confidence interval (CI). In addition, the NLS rate was analyzed using Kaplan–Meier survival analysis, as well as the log-rank test. Values were considered statistically significant when *P* < 0.05.

## Results

### Baseline Data of the BA Study Cohort

From February 2016 to May 2017, 422 type III BA patients were enrolled. The baseline characteristics at the time of KPE are presented in Table 1. The mean age was around 2 months, and the numbers of male and female patients were comparable. As expected, BA patients manifested hepatocellular damage and signs of obstructive jaundice with elevated DBIL, bile acids, and GGT. Approximately one-third of children were infected with CMV. A total of 51 extrahepatic malformations were detected in 47 patients (11.1%), including cardiac defects (38 patients, 9.0%), intestinal malformation (10 patients, 2.4%), splenic malformation (two patients, 0.5%), and visceral inversion (one patient, 0.2%). To improve the insufficient bile flow, 271 patients (64.2%) were administered glucocorticoids after KPE. The time to initial glucocorticoid treatment ranged from 5 to 60 days (median = 27 days) after KPE. Among the population with glucocorticoids, a good bile drainage occurred in 95 patients (30.06%), 135 patients (49.81%), 136 patients (50.18%), and 116 patients (42.80%) at 3, 6, 12, and 24 months after KPE, respectively. In total, the rate of JC climbed to the highest (59.5%) at 6 months after KPE and then fell slightly (52.4%) at 2 years after KPE. A total of 239 patients (56.6%) of BA patients survived with native liver; the rest comprised those with liver transplantation (18.7%) and death (24.6%) at 2 years after KPE. During the perioperative period, a total of three patients (0.7%) passed away. One patient who underwent KPE at the age of 136 days died of liver failure. Another patient who underwent KPE at the age of 45 days died of gastrointestinal bleeding due to portal hypertension. A third patient at day 38 after KPE suffered from intestinal obstruction and abnormal coagulation and died after her parents insisted on giving up treatment.

**Table 1:** Baseline characteristics of the biliary atresia study cohort (*n* = 422).

**Variable**	**Value**
Age (d) ($\bar{x}$ ± s)	62.9 ± 18.4
Female, *n* (%)	218 (51.7)
Weight (kg) ($\bar{x}$ ± s)	4.7 ± 0.8
Laboratory examinations ($\bar{x}$ ± s)	
ALT (U/L)	102.3 ± 77.3
AST (U/L)	169.4 ± 123.5
ALB (g/L)	38.5 ± 5.3
TBIL (μmol/L)	160.5 ± 65.9
DBIL (μmol/L)	109.6 ± 96.7
GGT (U/L)	682.8 ± 554.3
APRI	1.728 ± 1.611
Hb (g/L)	98.9 ± 16.3
INR	1.0 ± 0.2
TBA (μmol/L)	145.3 ± 87.5
Extrahepatic malformation, *n* (%)	47 (11.1)
CMV infection, *n* (%)	139 (32.9)
Glucocorticoid, *n* (%)	271 (64.2)
1-month JC, *n* (%)	30 (7.1)
3-months JC, *n* (%)	193 (45.7)
6-months JC, *n* (%)	251 (59.5)
1-year JC, *n* (%)	250 (59.2)
2-years JC, *n* (%)	221 (52.4)
1-year NLS, *n* (%)	329 (78.0)
2-years NLS, *n* (%)	239 (56.6)
2-years LT, *n* (%)	79 (18.7)
2-years death, *n* (%)	104 (24.6)

*ALB, albumin; ALT, alanine aminotransferase; APRI, aspartate aminotransferase-to-platelet ratio index; AST, aspartate aminotransferase; CMV, cytomegalovirus; DBIL, direct bilirubin; GGT, γ-glutamyl transpeptidase; Hb, hemoglobin; INR, international normalized ratio; TBA, total bile acid; TBIL, total bilirubin*.

### Characteristics of Perioperative Complications in the BA Study Cohort

**Figure 1:**
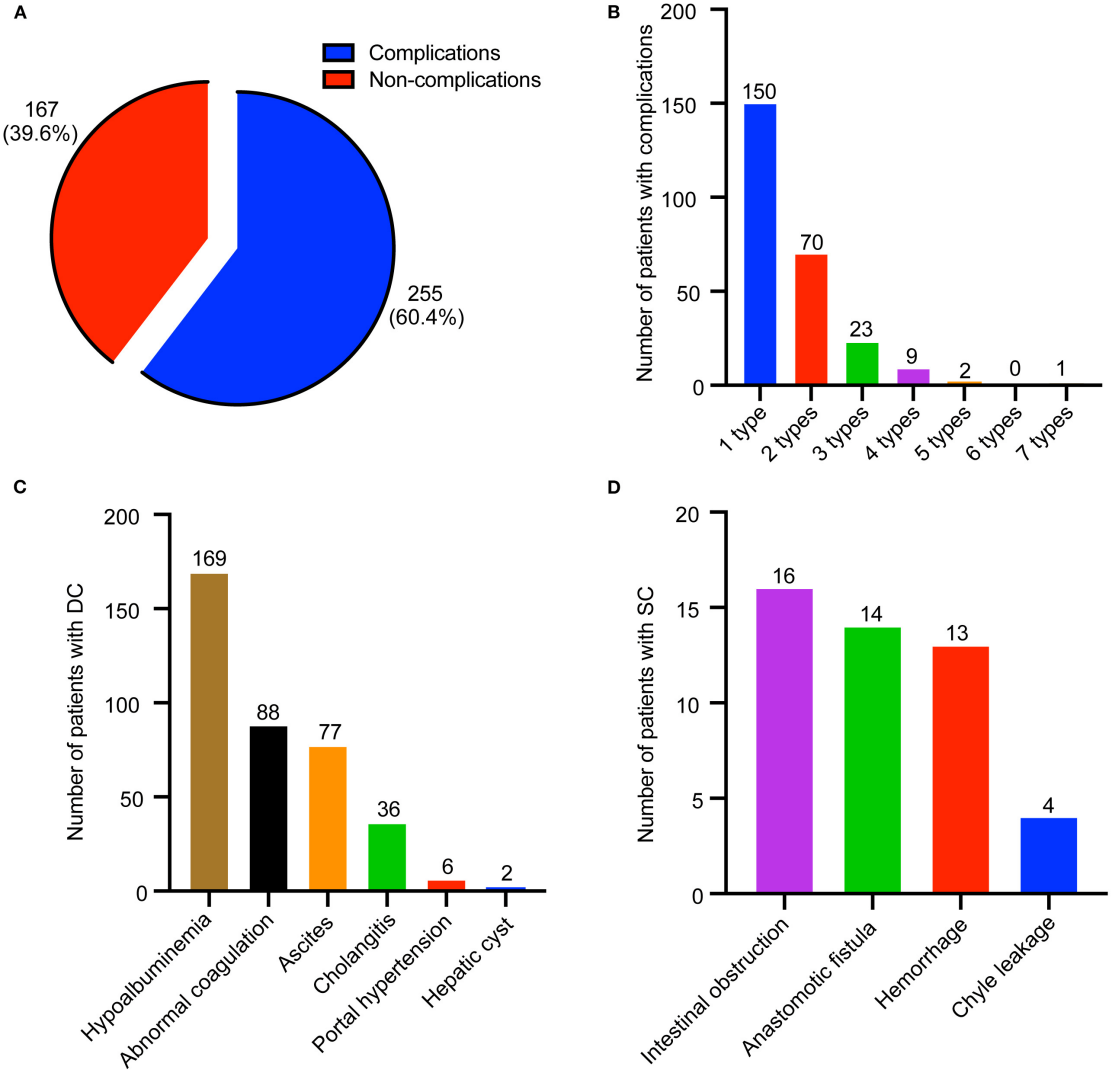
Distribution of perioperative complications in biliary atresia (BA) patients. **(A)** Composition of patients with or without complications. **(B)** Distribution of patients with different type of complications. **(C)** Distribution of disease-related complication (DC). **(D)** Distribution of surgical complication (SC).

In total, 255 patients (60.4%) had perioperative complications (Figure 1A). Two hundred forty-three patients (57.6%) suffered from one to three different types of complications per patient (Figure 1B), and only 12 patients (2.8%) suffered from four to seven different types of complications per patient. Furthermore, perioperative complications were divided into DCs and SCs; 246 patients (58.3%) had DCs, which mainly consisted of hypoalbuminemia, abnormal coagulation, ascites, and cholangitis (Figure 1C). However, only 29 patients (6.9%) suffered from SCs, which included intestinal obstruction, anastomotic fistula, hemorrhage, and chyle leakage (Figure 1D).

### Comparison Between BA Patients With and Without Perioperative Complications

To understand the difference between BA patients with and without perioperative complications, we compared their characteristics from the basic information, malformation, laboratory examination, treatment, and prognosis (Table 2). The results showed that patients with perioperative complications had lower serum ALB level, but higher APRI and INR levels when compared with those without perioperative complications (ALB, *P* < 0.05; APRI, *P* < 0.01; INR, *P* < 0.05). Moreover, perioperative complications were correlated with glucocorticoid administration (*P* = 0.002); less glucocorticoids were administered in the patients with perioperative complications (odds ratio = 0.518, 95% CI = 0.340–0.791). We focused on the correlation between perioperative complication and prognosis (NLS), and no significant differences were observed between the two groups (*P* > 0.05).

**Table 2:** Matched factors of biliary atresia patients with perioperative complications or not.

**Variable**	**Perioperative complications**		***P* value**
	**No (*n* = 167)**	**Yes (*n* = 255)**	
Age, d ($\bar{x}$ ± s)	62.5 ± 17.6	63.2 ± 19.0	0.700
Female, *n* (%)	82 (49.1)	136 (53.3)	0.395
Weight, kg ($\bar{x}$ ± s)	4.7 ± 0.8	4.7 ± 0.8	0.475
Laboratory examination ($\bar{x}$ ± s)			
ALT (U/L)	107.0 ± 71.9	99.2 ± 80.6	0.310
AST (U/L)	158.8 ± 120.0	176.4 ± 125.6	0.151
ALB (g/L)	39.3 ± 4.5	38.1 ± 5.7	**0.021**
TBIL (μmol/L)	160.0 ± 91.5	160.9 ± 41.7	0.899
DBIL (μmol/L)	102.1 ± 31.4	114.5 ± 121.6	0.196
GGT (U/L)	668.0 ± 550.8	692.5 ± 557.6	0.658
APRI	1.463 ± 1.208	1.910 ± 1.805	**0.002**
Hb (g/L)	100.3 ± 18.1	98.0 ± 15.0	0.162
INR	0.974 ± 0.195	1.024 ± 0.214	**0.016**
TBA (μmol/L)	143.9 ± 107.8	146.3 ± 71.4	0.783
Extrahepatic malformation, *n* (%)	19 (11.4)	28 (11.0)	0.899
CMV infection, *n* (%)	54 (32.3)	85 (33.3)	0.831
Glucocorticoid, *n* (%)	122 (73.1)	149 (58.4)	**0.002**
1-month JC, *n* (%)	13 (7.8)	17 (6.7)	0.662
3-months JC, *n* (%)	70 (41.9)	123 (48.2)	0.203
6-months JC, *n* (%)	92 (55.1)	159 (62.4)	0.137
1-year JC, *n* (%)	92 (55.1)	158 (62.0)	0.160
2-years JC, *n* (%)	84 (50.3)	137 (53.7)	0.491
1-year NLS, *n* (%)	131 (78.4)	198 (77.6)	0.847
2-years NLS, *n* (%)	96 (57.5)	143 (56.1)	0.776
2-years LT, *n* (%)	32 (19.2)	47 (18.4)	0.851
2-years death, *n* (%)	39 (23.4)	64 (25.1)	0.683

*ALB, albumin; ALT, alanine aminotransferase; APRI, aspartate aminotransferase-to-platelet ratio index; AST, aspartate aminotransferase; CMV, cytomegalovirus; DBIL, direct bilirubin; GGT, γ-glutamyl transpeptidase; Hb, hemoglobin; INR, international normalized ratio; JC, jaundice clearance; LT, liver transplantation; NLS, native liver survival; TBA, total bile acid; TBIL, total bilirubin. P values less than 0.05 were presented in bold*.

### Predictors for 2-Years Native Liver Survival of BA

**Figure 2:**
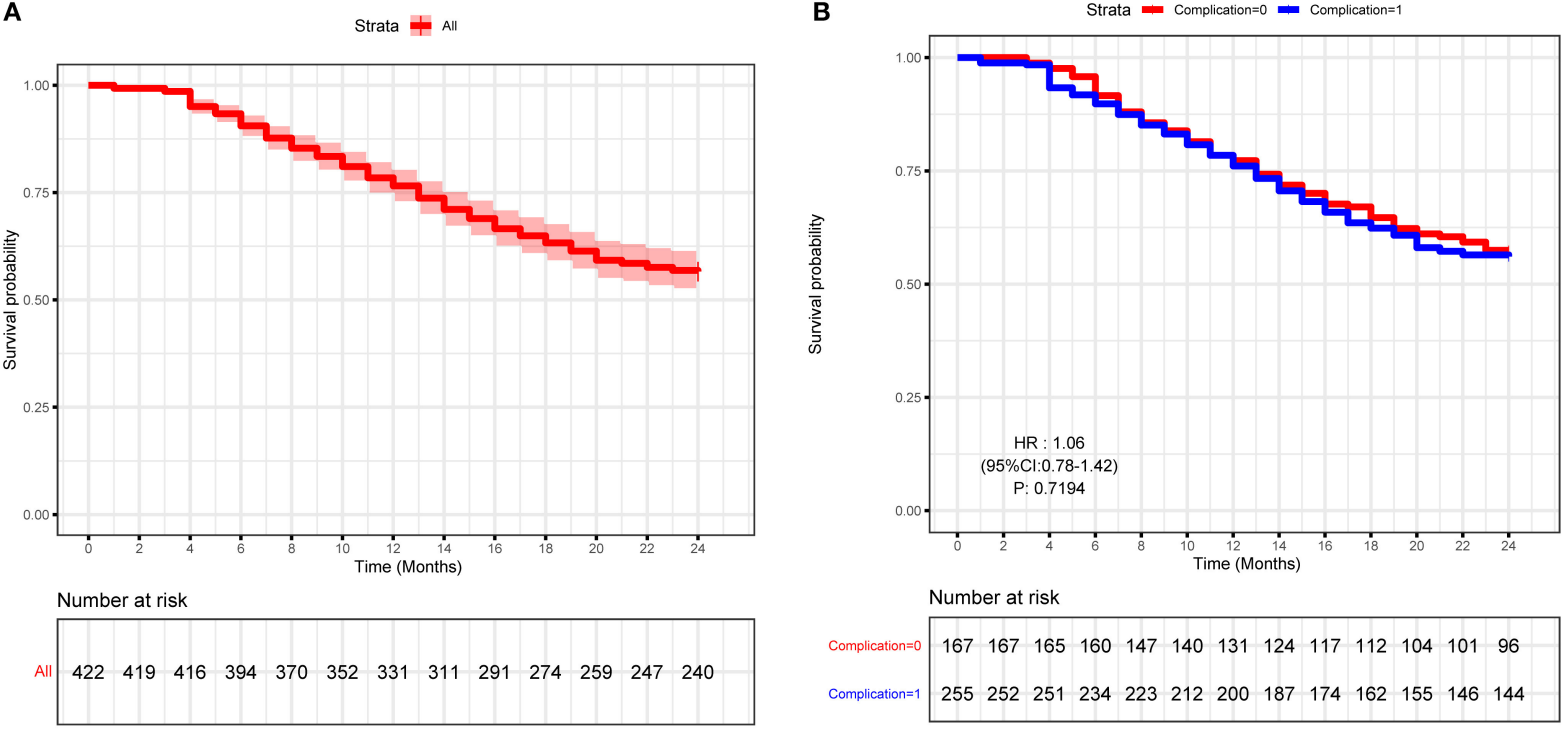
Kaplan–Meier curve of the study cohort of biliary atresia (BA) patients. **(A)** Kaplan–Meier curve of 422 type III BA patients. **(B)** Kaplan–Meier curve of BA patients with or without perioperative complications.

The survival curve of 2-years NLS is shown in Figure 2A. In this study, NLS was compared based on the presence of perioperative complication; Kaplan–Meier survival analysis also showed that BA patients with complications, and those without had comparable 2-years NLS (*P* = 0.551, Figure 2B). Therefore, perioperative complication was not a predictor for the 2-years NLS in BA.

**Figure 3:**
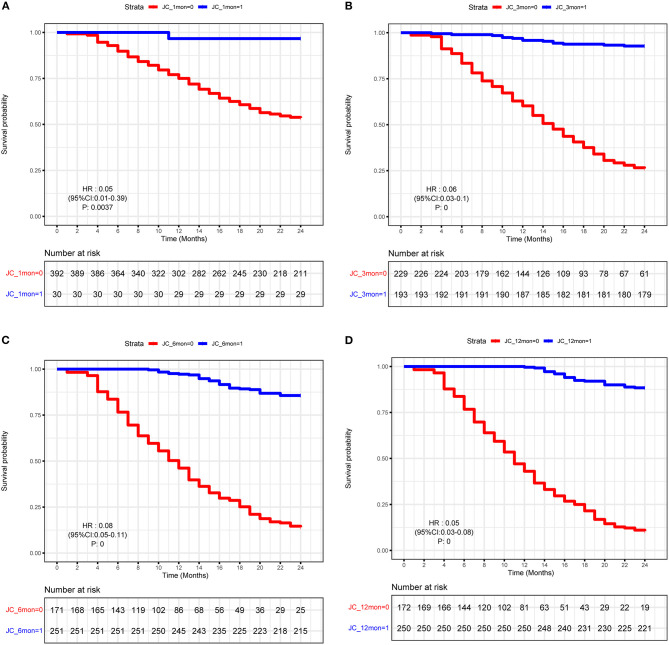
Kaplan–Meier curve of biliary atresia (BA) patients with or without JC. **(A)** 1-month JC. **(B)** 3-months JC. **(C)** 6-months JC. **(D)** 12-months JC.

To further explore the crucial factors that affected the 2-years NLS in BA, we first performed the univariate Cox regression analysis (Table 3). To avoid missing potential risk factors, we set the *P* threshold at 0.2. The results revealed that age, weight, APRI, glucocorticoid, and JC (1, 3, 6, and 12 months) were potential risk factors for 2-years NLS. Subsequently, four prediction models were established by the combination of age, weight, APRI, glucocorticoid, and JC at different time points after KPE (Table 4). The results showed JC was an independent protective factor for 2-years NLS (1-month JC, *P* = 0.008, HR = 0.070; 3-months JC, *P* = 0.000, HR = 0.060; 6-months JC, *P* = 0.000, HR = 0.074; 12-months JC, *P* = 0.000, HR = 0.051). To validate the appropriate model in predicting 2-years NLS, we compared concordance indexes (C indexes) and found they were 0.62, 0.76, 0.81, and 0.83 in the prediction models with 1-month JC, 3-months JC, 6-months JC, and 12-months JC, respectively (Table 4). Given the timeliness of the prediction model, we believed that the model with 6-months JC was the most effective in predicting 2-years NLS (C index: 0.81). Kaplan–Meier survival analysis also supported the above findings that BA patients with JC had a remarkably higher 2-years NLS than those with jaundice (*P* < 0.01) (Figure 3).

**Table 3:** Univariate Cox regression analysis of predictors for 2-years native liver survival in biliary atresia (*n* = 422).

**Variable**	**2-years NLS**		
	***P*-value**	**HR**	**95% CI**
Age	0.161	1.006	0.998–1.014
Gender	0.541	1.095	0.819–1.462
Weight	0.012	1.251	1.050–1.490
ALT	0.556	0.999	0.998–1.001
AST	0.378	1.000	0.999–1.002
ALB	0.870	1.002	0.974–1.032
TBIL	0.510	0.999	0.997–1.002
DBIL	0.498	0.999	0.996–1.002
GGT	0.751	1.000	1.000–1.000
Hb	0.848	1.001	0.992–1.009
INR	0.888	1.046	0.560–1.952
TBA	0.791	1.000	0.999–1.002
APRI	0.029	1.095	1.009–1.189
Extrahepatic malformation	0.439	0.825	0.507–1.342
CMV infection	0.824	0.965	0.708–1.316
Complication	0.725	1.055	0.784–1.420
Glucocorticoid	0.000	1.992	1.419–2.797
1-month JC	0.004	0.055	0.008–0.395
3-months JC	0.000	0.059	0.034–0.102
6-months JC	0.000	0.079	0.054–0.114
1-year JC	0.000	0.054	0.036–0.081

*ALB, albumin; ALT, alanine aminotransferase; APRI, aspartate aminotransferase-to-platelet ratio index; AST, aspartate aminotransferase; CI, confidence interval; CMV, cytomegalovirus; DBIL, direct bilirubin; GGT, γ-glutamyl transpeptidase; Hb, hemoglobin; HR, hazard ratio; INR, international normalized ratio; JC, jaundice clearance; LT, liver transplantation; NLS, native liver survival; TBA, total bile acid; TBIL, total bilirubin*.

**Table 4:** Multivariate Cox regression analysis of predictors for 2-years native liver survival in biliary atresia (*n* = 422).

**Variable**	**2-years NLS**			**C index**
	**Adjusted *P*-value**	**Adjusted HR**	**Adjusted 95% CI**	
**Model 1**				
Age	0.629	1.003	0.992–1.014	0.62
Weight	0.050	1.267	1.000–1.606	
APRI	0.206	1.057	0.970–1.151	
Glucocorticoid	0.003	1.697	1.192–2.416	
1-month JC	0.008	0.070	0.010–0.504	
**Model 2**				
Age	0.452	0.996	0.985–1.007	0.76
Weight	0.098	1.230	0.962–1.572	
APRI	0.098	1.072	0.987–1.163	
Glucocorticoid	0.869	1.031	0.720–1.476	
3-months JC	0.000	0.060	0.034–0.105	
**Model 3**				
Age	0.535	0.997	0.986–1.007	0.81
Weight	0.016	1.351	1.057–1.726	
APRI	0.165	1.065	0.974–1.164	
Glucocorticoid	0.428	0.862	0.596–1.245	
6-months JC	0.000	0.074	0.050–0.110	
**Model 4**				
Age	0.936	1.000	0.989–1.011	0.83
Weight	0.117	1.218	0.951–1.560	
APRI	0.042	1.098	1.004–1.202	
Glucocorticoid	0.363	0.843	0.584–1.218	
1-year JC	0.000	0.051	0.033–0.078	

*APRI, aspartate aminotransferase-to-platelet ratio index; JC, jaundice clearance; NLS, native liver survival*.

## Discussion

To our knowledge, this is the first large study that analyzed perioperative complications after KPE for patients with type III BA. In our study, the incidence and mortality rates of perioperative complications were 60.4 and 0.7%, respectively, which were much lower compared to the findings that were presented previously (96.8 and 2%, respectively) (14, 21). This may be related to the fact that our institution, the largest BA center in China, is highly experienced in performing KPE. In a multicenter study in Europe, it was also observed that high caseload benefited outcomes (22, 23). Most patients (243, 57.6%) had one to three different types of complications, and DCs were the most prevalent (246, 58.3%). The most common type of DCs was hypoalbuminemia, followed by abnormal coagulation, ascites, cholangitis, portal hypertension, and hepatic cysts. The incidence of perioperative cholangitis was low (8.5%), which was potentially due to the short perioperative observation period of 30 days. Notably, recurrent cholangitis can lead to a lower NLS (24). Patients who experience a fever and worsening jaundice need to be vigilant of cholangitis (24, 25), and the use of antireflux valves, jejunal Roux-en-Y loop and antibiotics may help to reduce the occurrence of cholangitis (25, 26). In a recent study in 2015, it was shown that SCs affected 41% of 153 patients (27), which was much higher than our results (6.9%). In our study cohort, we did observe hemorrhage, anastomotic fistula, intestinal obstruction, and chyle leakage; however, hernia and abscess complications that have been reported in previous studies were not observed (27). Our data showed that intestinal complications (intestinal obstruction and anastomotic fistula) were the most frequent SCs, which was consistent with the findings presented in other reports (14). After KPE, patients with acute abdominal pain must immediately and carefully be assessed for intestinal complications. In order to reduce intestinal complications, care must be taken to accurately close the mesenteric defect and to not twist the intestinal loop during KPE (28). The incidence of hemorrhage was 3.1% without massive bleeding or hemorrhagic shock, which was lower than the findings reported previously (5%) (27). Correction of coagulation and protection of blood vessels can reduce hemorrhage. In this study, serum ALB concentration, INR, and APRI had a significant change in BA patients with perioperative complications when compared with those without complications, which indicated that liver synthetic functions or fibrosis may be risk factors for disease-related perioperative complications in BA.

In previous studies, it has been shown that the short-term (1–3 years) NLS rate of BA ranged from 20.3 to 75.8% (10). Consistent with this, in our study, the 1-year and 2-years NLS rates were 78.0 and 56.6%, respectively. Although perioperative complications were common in BA patients, they were not predictors for 2-years NLS, which suggested perioperative complications had no impact on the BA prognosis. However, JC was considered a crucial predictor for improved NLS (11, 29–32). In a large study in Japan, it was demonstrated that TBIL <17.1 μmol/L at 3 months post-KPE predicted an improved NLS (33). Moreover, in another multicenter study in the United States, it was shown that TBIL <34.2 μmol/L at 3 months after KPE was a predictor for improved NLS (34). In this study, the JC (TBIL <20 μmol/L) rate was also highly predictive for 2-years NLS. Furthermore, we evaluated the accuracy of prediction models by comparing C index and found the prediction model with 6-months JC was more appropriate than other models with 1-month JC, 3-months JC, and 12-months JC, which may be explained by the highest level at 6 months after KPE. Taken together, these findings suggested that JC after KPE resulted in a good bile flow with better NLS.

Treatment with adjuvant steroids after KPE has previously been used to assist restoring bile flow and anti-inflammation; however, the effects of steroids on BA outcome remain controversial (35, 36). In several studies, no benefits of postoperative steroids on prognosis were observed (36, 37), whereas other studies showed that treatment with adjuvant steroids after KPE may improve short-term (≤1 year) JC. In this study, our results revealed that patients treated with adjuvant steroids initially suffered from no postoperative bile drainage and also had a higher risk obtaining a low 2-years NLS (HR = 1.992, 95% CI = 1.419–2.797, *P* = 0.000). The paradoxical effects of the use of steroids on the prognosis of BA remain to be elucidated.

In this study, the average age at KPE was 62.9 ± 18.4 days, which was similar to that published in international journals (38). A young age, especially <60 days old at KPE, has been widely, although not universally, linked to improved outcome in BA, which may be explained by the milder liver fibrosis in younger patients (16, 39–41). However, Song et al. (42) demonstrated that BA patients younger than 40 days had a poorer NLS rate and that, in these patients, severe liver fibrosis may account for the reverse consequence (35, 39). Interestingly, our results showed that age at KPE had no significant impact on prognosis of BA. Despite the controversy, it has been advised that infants with cholestasis need to be evaluated and appropriately managed on time and that when BA is diagnosed KPE should be performed as soon as possible (43–45).

Aspartate aminotransferase-to-platelet ratio index reflects portal fibrogenesis and could diagnose liver fibrosis in BA (40, 46). Ihn et al. (29) demonstrated that APRIs at month 4, GGT at month 5, and JC at month 6 after KPE were independent risk factors for NLS. Grieve et al. (47) also supported the role of APRI at KPE in predicting NLS using univariate analysis. Our results showed that APRI at KPE had significance in predicting NLS without considering other factors, but exerted a modest effect in the prediction model for NLS. Therefore, monitoring the change of APRI may be valuable in predicting NLS in BA.

Studies in the United Kingdom and Germany (48, 49) demonstrated that approximately 10% of BA patients were infected with CMV. However, a much higher incidence of CMV infection (34–69%) was reported in Brazil, Sweden, Pakistan, China, and South Africa (50–54). In the current study, CMV infection was present in 139 patients (32.9%). The varied incidence of CMV infection may be explained by the different racial background (55). Hepatotropic viral infections have been proposed as a causative mechanism of action for bile duct and immune destruction (56). Liver T-cell responses to CMV and deficiency of regulatory T cells in BA aggravated inflammation and autoreactivity, which potentially allowed for exaggerated bile duct injury (57). Zani et al. (49) found that BA patients with positive CMV IgM had lower JC and NLS. However, our results indicated that CMV infection had little impact on prognosis, which was consistent with the findings presented in two German studies (48, 58). The above results suggested a controversial role of CMV infection in BA. In line with the data of Zani et al. (49), our results also indicated that CMV-positive BA patients were significantly older than those who were CMV negative (CMV negative: 60.69 ± 18.43 days; CMV positive: 67.36 ± 17.70 days; *P* < 0.001), implying that CMV infection was simply a secondary phenomenon rather than a factor involved in the etiology of BA. Therefore, our data demonstrated that CMV may not play a major role in the pathogenesis of BA, and further studies are needed in the future.

This was a single-center retrospective study, which was considered a limitation. Nevertheless, strict inclusion and exclusion criteria were adopted, and all surgeries were performed by the same surgical team, which would increase the consistency and could be regarded as an advantage. A 2-years NLS rate represented a short-term prognosis when compared with the 10–20-years results that have been reported in other studies. However, in this study, multivariate analysis models were applied, and important predictors for prognosis were identified, including age, weight, APRI, glucocorticoids, and JC. Notwithstanding these limitations, this study, in which a large sample size was used, comprehensively revealed the characteristics of BA perioperative complications and prognosis, thereby providing the scope for future research in perioperative management.

In conclusion, perioperative complication was common in BA, but was not a predictor for 2-years NLS. However, 6-months JC after KPE was an independent protective factor for 2-years NLS. Therefore, monitoring the dynamic change of serum TBIL and taking the corresponding treatments after KPE may be crucial in improving the survival rate of native liver in BA.

## Data Availability Statement

All datasets generated for this study are included in the article/supplementary material.

## Ethics Statement

The studies involving human participants were reviewed and approved by Children's Hospital of Fudan University. Written informed consent to participate in this study was provided by the participants' legal guardian/next of kin. Written informed consent was obtained from the minor(s)' legal guardian/next of kin for the publication of any potentially identifiable images or data included in this article.

## Author Contributions

SZ and RD conceived and designed the study. MD and JW collected the clinical data, performed data analysis, and wrote the paper. YT and JJ offered the assist in data collection. GC, YH, and ZS reviewed and edited the manuscript. All authors read and approved the manuscript.

## Conflict of Interest

The authors declare that the research was conducted in the absence of any commercial or financial relationships that could be construed as a potential conflict of interest.
